# Supplementary material for: Dynamic Profile of the Yak Mammary Transcriptome during the Lactation Cycle
Source: Animals (Basel). 2023 May 22;13(10):1710. doi: 10.3390/ani13101710 (PMC10215676; doi:10.3390/ani13101710)
Supplement: Supplementary file 1 [file animals-13-01710-s001.zip › animals-2257037-supplementary figures.pdf]

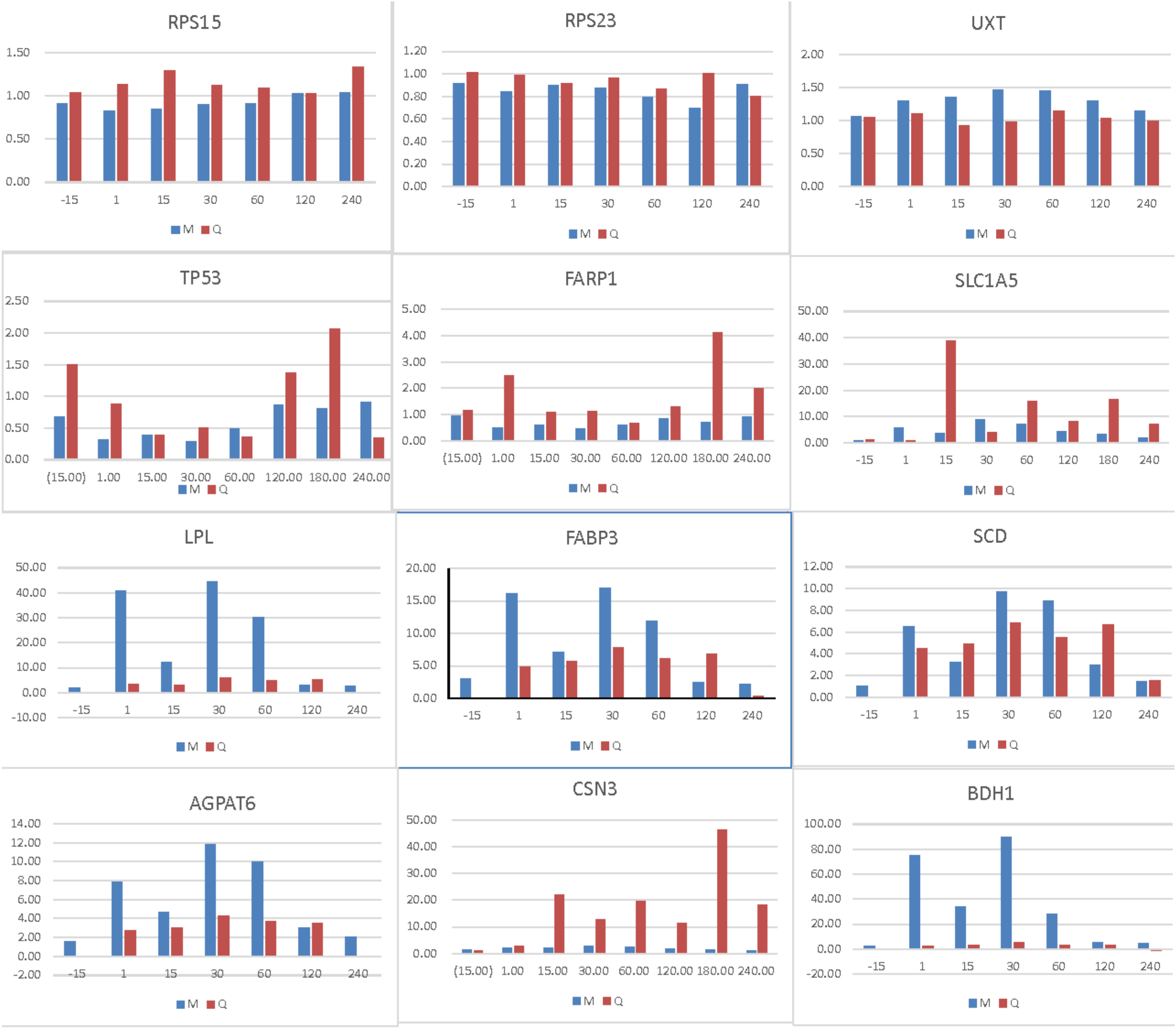

Supplementary Figure S1. Comparison of selected genes of Microarray (M: blue) and qPCR (Q: red) data in yak mammary gland during lactation.

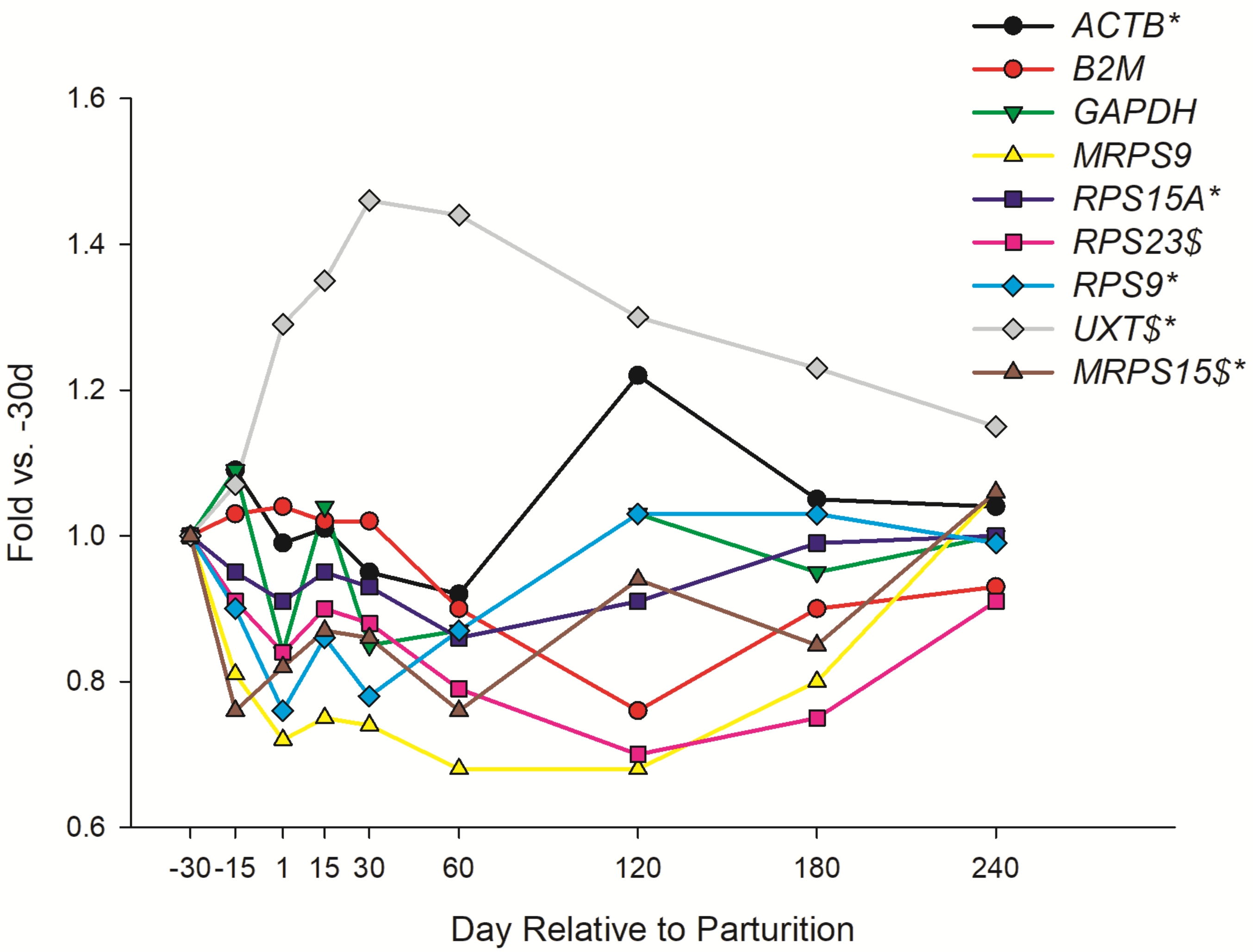

Suppl. Figure 2. Evaluation of the transcription pattern as detected by microarray on previously assessed potential internal control genes for RTqPCR. $ indicate prior reference genes used to normalize RTqPCR data from the same samples used in the present manuscript (Jiang et al., 2016).* indicate transcripts that were statistfially affected with a FDR<0.05 by day relative to parturition in yak mammary tissue in the current experiment (see Suppl. File 1)
